# Surgically Managed Orbital Tumors: A Case Series from a Referral Center in Iran

**DOI:** 10.18502/jovr.v18i2.13178

**Published:** 2023-04-19

**Authors:** Arman Mashayekhi

**Affiliations:** ^1^Ophthalmology Department, Mayo Clinic, Jacksonville, FL, USA

The spectrum of orbital tumors is wide, including a variety of benign and malignant neoplasms. The reported incidence of different orbital tumors depends on several variables related to the geographic location of the study center and many other factors related to the type of study performed.

The effect of geographic location is well illustrated by the reported incidence of orbital retinoblastoma. For example, in a study on pediatric orbital tumors from Turkey, secondary orbital retinoblastoma accounted for 33% of cases, a rate much higher than that reported from more economically developed countries.^[1]^

The selection criteria of the study affect the reported incidence of orbital tumors in many ways. Whereas some studies include all clinically, radiographically, and pathologically diagnosed orbital space occupying tumors and pseudotumors,^[2]^ others report only pathologically proven cases.^[3]^

The age distribution of the included patients will have significant effects on the results of the study as the types of orbital tumors seen in children are drastically different from those reported in adults. In studies reported from the United States on orbital tumors in children the most common tumors are benign cystic and vascular lesions but lymphoproliferative lesions are the most common tumors reported in older adults.^[4,5,6]^

The characteristics of the reporting facility where the study was performed can also impact the results of the study. In a study from a large ocular oncology center, of the secondary orbital tumors, 29% were orbital extension of uveal melanoma and 13% were orbital extension of conjunctival melanoma.^[2]^ Majority of these patients were referred for treatment of the primary uveal or conjunctival melanoma and were found on further investigation to have orbital involvement. Similarly, in another study from a comprehensive cancer center in the United States, the most common orbital tumors were found to be secondary tumors (26% of cases).^[7]^

In this issue of the *Journal of Ophthalmic and Vision Research*, Bagheri and coworkers report the results of a retrospective study on space-occupying lesions of the orbit from a referral ophthalmology center in Tehran, Iran.^[8]^ Their study includes orbital tumors that were managed over a 12-year period between 2008 and 2020 and had a definite histopathological diagnosis.

The requirement for a histopathological diagnosis is both a strength and a limitation of this study. On the one hand, diagnosis of orbital space-occupying lesions based on histopathological evaluation is more accurate than that based on clinical and radiological studies alone. On the other hand, this requirement leads to exclusion of many orbital space-occupying lesions that do not undergo surgical resection. For instance, a small orbital tumor with radiological features consistent with a cavernous hemangioma incidentally discovered in an asymptomatic patient is more likely to be observed and therefore would not have been included in the study. In the study by Shields and coworkers, only half of the presumed cavernous hemangiomas of the orbit underwent surgical removal.^[2]^

The study by Bagheri and coworkers includes patients of all ages, from 1 to 94 years, but is more skewed toward younger patients with a mean age of 31 years and with only 12% of the patients older than 60 years. It is therefore no surprise that dermoid cysts are the most common tumors in this study (one-third of all cases) and orbital lymphoma comprises only 4% of all the orbital tumors. In comparison, in the study by Shields and coworkers, only 6% of the tumors were cystic and 10% were lymphoid or leukemic.^[2]^

The geographic location of the medical center in which the patients were treated by Dr Bagheri and his coworkers, the capital city of Tehran, could have affected the results of their study. Although as a tertiary referral medical facility, the Labbafinejad Medical Center provides medical care to patients from different parts of the country, it is quite likely that most of their patients were from Tehran or surrounding areas where there is easier access to high-quality medical care.

A noteworthy finding of the study by Bagheri and coworkers is the absence of any cases of orbital invasion by retinoblastoma. Although I am not aware of any older studies from Iran, in an older study from Turkey including patients seen between 1963 and 1993, about one-third of pediatric orbital tumors were secondary orbital invasion by retinoblastoma.^[1]^ Similarly, in a study that included patients managed in Saudi Arabia in the 1980s, 32% of pediatric orbital tumors proved to be orbital invasion of retinoblastoma.^[9]^ A more recent study from a tertiary eye center in Saudi Arabia including pediatric patients managed between 2000 and 2013 does not show any cases of orbital retinoblastoma.^[10]^ The absence of orbital invasion by retinoblastoma in the studies by Bagheri and coworkers and by Alkatan and coworkers most likely are due to improved medical care in the areas served by the respective treating medical facilities allowing timely diagnosis and treatment of retinoblastoma.

The study by Dr Bagheri and his colleagues provides important information on the distribution of surgically resected orbital tumors in Iran and the authors should be commended for their efforts. Further studies on this subject from medical centers in other parts of the country are necessary to provide a more comprehensive view of the distribution of orbital tumors in the country.

## References

[B1] Günalp I, Gündüz K (1995). Pediatric orbital tumors in Turkey. Ophthalmic Plast Reconstr Surg.

[B2] Shields JA, Shields CL, Scartozzi R (2004). Survey of 1264 patients with orbital tumors and simulating lesions: The 2002 Montgomery Lecture, part 1. Ophthalmology.

[B3] Montano N, Lauretti L, D'Alessandris QG, Rigante M, Pignotti F, Olivi A, et al

[B4] Bullock JD, Goldberg SH, Rakes SM (1989). Orbital tumors in children. Ophthalmic Plast Reconstr Surg.

[B5] Castillo BV Jr, Kaufman L (2003). Pediatric tumors of the eye and orbit. Pediatr Clin North Am.

[B6] Demirci H, Shields CL, Shields JA, Honavar SG, Mercado GJ, Tovilla JC (2002). Orbital tumors in the older adult population. Ophthalmology.

[B7] Shinder R, Al-Zubidi N, Esmaeli B (2011). Survey of orbital tumors at a comprehensive cancer center in the United States. Head Neck.

[B8] Bagheri A, Ashtar-nakhaie P, Aletaha M, Kheiri B, Veisi A

[B9] Johnson TE, Senft SH, Nasr AM, Bergqvist G, Cavender JC (1990). Pediatric orbital tumors in Saudi Arabia. Orbit.

[B10] Alkatan HM, Al Marek F, Elkhamary S (2019). Demographics of pediatric orbital lesions: A tertiary eye center experience in Saudi Arabia. J Epidemiol Glob Health.

